# Necrotising fasciitis of the shoulder in association with rheumatoid arthritis treated with etanercept: a case report

**DOI:** 10.1186/1752-1947-4-367

**Published:** 2010-11-17

**Authors:** Andrew Smyth, Diarmaid D Houlihan, Helen Tuite, Catherine Fleming, Thomas A O'Gorman

**Affiliations:** 1 Department of Gastroenterology, Galway University Hospitals, Galway, Ireland; 2 Department of Medicine, School of Medicine & Health Sciences, National University of Ireland, Galway, Ireland; 3 Department of Infectious Diseases, Galway University Hospitals, Galway, Ireland

## Abstract

**Introduction:**

Necrotising fasciitis is a severe infection characterised by the fulminant destruction of tissue with associated systemic signs of sepsis and toxicity. Etanercept is a fully human fusion protein that inhibits tumor necrosis factor and the inflammatory cascade. It is effective in the treatment of many disorders but concerns regarding severe life threatening infections have been raised in multiple reports.

**Case presentation:**

We present the case of a 39-year-old Caucasian man, who presented with sudden onset of severe and progressive neck and left shoulder pain, with a two-year history of seronegative rheumatoid arthritis treated with azathoprine and etanercept. On examination the left side of his neck and his left shoulder were oedematous, tender with an erythematous rash and his active range of movement was limited. Magnetic resonance imaging of his shoulder showed extensive oedema of the subcutaneous and intramuscular fat of the left lower neck consistent with fasciitis. He was treated medically and made a good recovery.

**Conclusion:**

Our patient, while having a pre-existing increased mortality risk, had a serious infection which responded well to optimum medical treatment without the need for surgery. As anti tumor necrosis factor agents are frequently associated with infection, including tuberculous infection, this case highlights the need for a high index of suspicion for other severe bacterial infections in patients on immunosuppressants.

## Introduction

Necrotising fasciitis (NF) is a severe infection characterised by the fulminant destruction of tissue with associated systemic signs of sepsis and toxicity. It affects the skin and fascia and is frequently associated with thrombosis of blood vessels and aggressive extension along fascial planes is associated with a high mortality rate. In this report, we describe the case of a man with rheumatoid arthritis who developed NF of his left shoulder. There are no previous reports of NF of the shoulder in association with etanercept treatment published to date.

## Case presentation

A 39-year-old Caucasian male presented with the sudden onset of severe and progressive neck and left shoulder pain, radiating down the left arm with decreased range of movement at the shoulder. He denied any preceding trauma. His past medical history was significant for a two-year history of seronegative rheumatoid arthritis treated with azathoprine and etanercept, the last dose of which had been administered three weeks prior.

On examination, he was guarding his left neck and shoulder. He was haemodynamically stable and febrile at 38.3°C. His left shoulder area was oedematous with an erythematous rash, and was diffusely tender both anteriorly and posteriorly. Passive range of movement was painful and active range of movement was very limited. Neurological and vascular examinations were normal. He had no clinical evidence of toxic shock syndrome.

Initial investigations revealed neutrophilia with an elevated CRP and blood cultures were subsequently negative. Chest radiograph and plain films of the left shoulder and cervical spine were normal. Differential diagnosis included septic arthritis, osteomyelitis and localized skin abscess. As a result, his physician commenced intravenous cefotaxime and immunosuppressant medications were immediately discontinued. Over the following 24 hours he remained febrile and developed a diffuse macular blanching rash over the left shoulder with a non-fluctuant boggy swelling in the left supraclavicular area. Antimicrobial therapy was changed to intravenous cefotaxime, metronidazole, vancomycin and ciprofloxacin. Serial examination of the shoulder showed persistent tenderness and progression of the erythematous macular rash with defined areas across the left shoulder area, but with no evidence of blistering, skin eruption or discharge.

**Figure 1:**
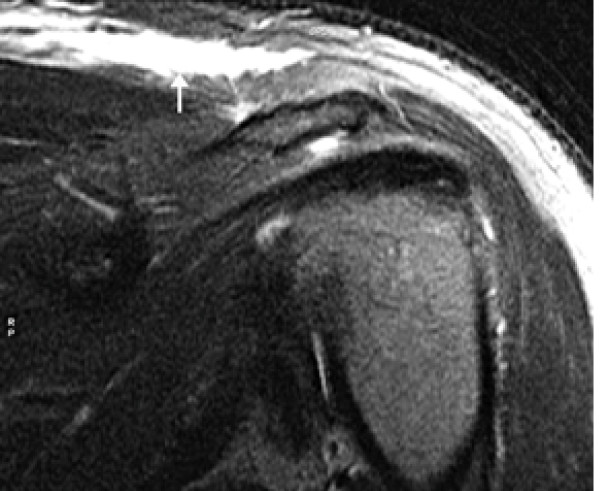
**Coronal oblique fat-suppressed post contrast MRI left shoulder (TR/TE 5670/75)**.

**Figure 2:**
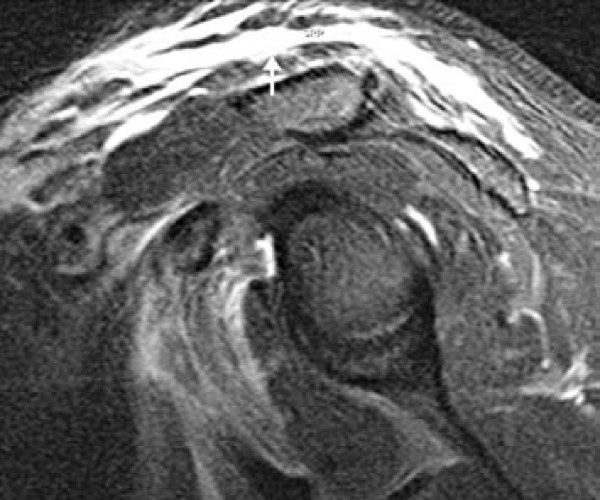
**Sagittal oblique fat-suppressed post contrast MRI left shoulder (TR/TE 5670/75)**. There is extensive subcutaneous oedema in the soft tissues superficial to the deltoid and rotator cuff muscles (arrow), consistent with left shoulder fascitis. The underlying supraspinatous tendon and muscles are normal.

**Figure 3:**
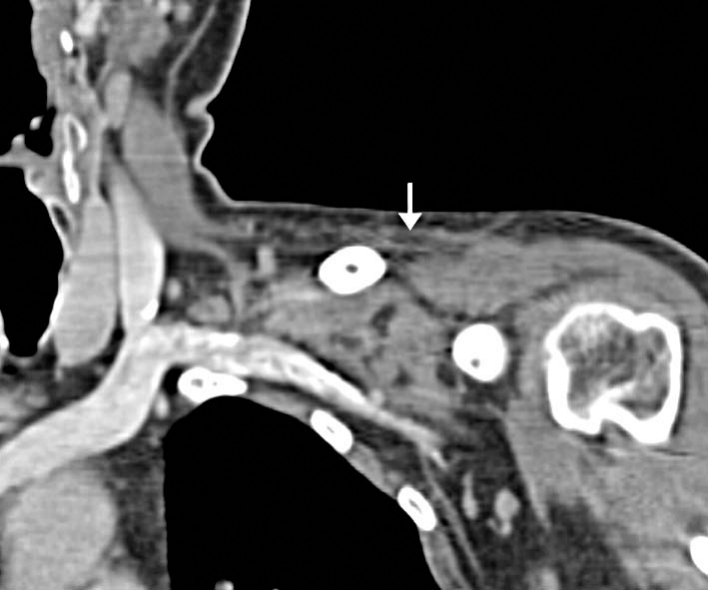
**Coronal CT scan post-intravenous contrast following completion of antibiotic therapy (kVp 120, modulated mA)**. There has been almost complete interval resolution of the subcutaneous oedema, with minimal residual inflammatory fat-stranding in the supra- and infra-clavicular spaces.

As a result of ongoing symptoms, an MRI with intravenous gadolinium contrast of the shoulder was performed and showed extensive oedema of the subcutaneous and intramuscular fat of the left lower neck, supraclavicular region, pectoralis major and teres minor muscles. There was no evidence of a discreet fluid collection or abnormality within the muscles or osseus structures. The appearances were felt to be consistent with extensive fasciitis (Figures 1 and 2). He was clinically improving, as a result medical and surgical reviewers agreed with antimicrobial consolidation to intravenous clindamycin, ciprofloxacin, flucloxacillin and benzylpenicillin with the caveat of close monitoring and surgical intervention at any sign of deterioration or progression. He clinically improved and inflammatory indices slowly normalised. He received a total of 14 days of intravenous antimicrobials followed by oral equivalents for a further 14 days. Follow up CT of his shoulder showed resolution of the inflammatory changes (Figure 3). He was closely followed up as an outpatient for a number of months after dismissal from the hospital with no evidence of recurrence.

## Discussion

Etanercept is a fully human fusion protein that inhibits tumor necrosis factor (TNF) and the inflammatory cascade, shown to be safe and effective in the management of patients with rheumatoid arthritis [1]. It has been shown to be associated with increased mortality [2] and with septic shock and disseminated intravascular coagulation [3]. Anti-TNF agents have a substantial risk of infection with *Mycobacterium tuberculosis *[4-6]. Although trials support safety of usage of etanercept [7], concerns regarding severe life threatening infections have been raised in multiple reports including the incidence of opportunistic infection [8].

NF can affect any fascial compartment of the body but higher rates of mortality are seen in patients with co-morbidities [9]. NF involvement of the shoulder area is rare. NF is most commonly associated with Group A Streptococcus infection, but other organisms have been implicated. As such, empiric treatment requires the use of agents targeted against these organisms. NF is a very serious infection and management is predominantly surgical as excision and debridement of infected tissue is the mainstay of management. Our patient's clinical presentation was atypical and by the time that full diagnosis was made he had clinically improved with medical management alone. Our patient did not undergo surgical management, but would have if there were any evidence of deterioration or progression.

Most patients who develop necrotising fasciitis have a predisposing immunocompromising condition or a local defect [10]. As there was no apparent trauma or inciting event, we believe that the predisposing factor to the development of the infection in this case was etanercept.

## Conclusion

Our patient, while having a pre-existing increased mortality risk, had a serious infection that responded well to optimum medical treatment. As anti-TNF agents are more frequently associated with tuberculous infection, this case highlights the need for a high index of suspicion for other severe bacterial infections in patients on immunosuppressants.

## Abbreviations

CRP: C-Reactive Protein; CT: Computed Tomography; MRI: Magnetic Resonance Imaging; NF: necrotising fasciitis; TNF: tumor necrosis factor.

## Consent

Written informed consent was obtained from the patient for publication of this case report and accompanying images. A copy of the written consent is available for review by the Editor-in-Chief of this journal.

## Competing interests

The authors declare that they have no competing interests.

## Authors' contributions

AS was responsible for the daily clinical management of the patient, preparation of manuscript, the literature review and submission of the manuscript. DH supervised daily management of our patient and edited the manuscript during preparation. HT provided a daily infectious disease evaluation of the patient and assisted in manuscript preparation. CF supervised the contribution of HT. TOG was the primary physician, who co-ordinated care of the patient. All authors read and approved the final manuscript.

## References

[B1] Bathon JM Martin RW Fleischmann RM Tesser JR Schiff MH Keystone EC Genovese MC Wasko MC Moreland LW Weaver AL Markenson J Finck BK A comparison of etanercept and methotrexate in patients with early rheumatoid arthritis N Engl J Med 2000 343 1586 1593 10.1056/NEJM200011303432201 11096165

[B2] Fisher CJ Jr Agosti JM Opal SM Lowry SF Balk RA Sadoff JC Abraham E Schein RM Bengamin E Treatment of septic shock with the tumor necrosis factor receptor:Fc fusion protein. The Soluble TNF Receptor Sepsis Study Group N Engl J Med 1996 334 1697 1702 10.1056/NEJM199606273342603 8637514

[B3] O'Dell JR Anticytokine therapy--a new era in the treatment of rheumatoid arthritis? N Engl J Med 1999 340 310 312 10.1056/NEJM199901283400411 9920958

[B4] Reddy JG Loftus EV Jr Safety of infliximab and other biologic agents in the inflammatory bowel diseases Gastroenterol Clin North Am 2006 35 837 855 10.1016/j.gtc.2006.09.008 17129816

[B5] Kavanagh PM Gilmartin JJ O'Donnell J O'Flanagan D Tumour necrosis factor-alpha and tuberculosis: guidance from the National TB Advisory Committee Ir Med J 2008 101 6 7 18369014

[B6] Askling J Fored CM Brandt L Baecklund E Bertilsson L Coster L Geborek P Jacobsson LT Lindblad S Lysholm J Rantapaa-Dahlqvist S Sazne T Romanus V Klareskog L Feltelius N Risk and case characteristics of tuberculosis in rheumatoid arthritis associated with tumor necrosis factor antagonists in Sweden Arthritis Rheum 2005 52 1986 1992 10.1002/art.21137 15986370

[B7] Schneeweiss S Setoguchi S Weinblatt ME Katz JN Avorn J Sax PE Levin R Solomon DH Anti-tumor necrosis factor alpha therapy and the risk of serious bacterial infections in elderly patients with rheumatoid arthritis Arthritis Rheum 2007 56 1754 1764 10.1002/art.22600 17530704

[B8] Kroesen S Widmer AF Tyndall A Hasler P Serious bacterial infections in patients with rheumatoid arthritis under anti-TNF-alpha therapy Rheumatology (Oxford) 2003 42 617 621 10.1093/rheumatology/keg263 12709536

[B9] Childers BJ Potyondy LD Nachreiner R Rogers FR Childers ER Oberg KC Hendricks DL Hardesty RA Necrotizing fasciitis: a fourteen-year retrospective study of 163 consecutive patients Am Surg 2002 68 109 116 11842952

[B10] Praba-Egge AD Lanning D Broderick TJ Yelon JA Necrotizing fasciitis of the chest and abdominal wall arising from an empyema J Trauma 2004 56 1356 1361 10.1097/01.TA.0000042157.00868.2E 15211151

